# Physiological ecology and functional traits of North American native and Eurasian introduced *Phragmites australis* lineages

**DOI:** 10.1093/aobpla/plt048

**Published:** 2013-10-24

**Authors:** Thomas J. Mozdzer, Jacques Brisson, Eric L. G. Hazelton

**Affiliations:** 1 Biology Department, Bryn Mawr College, Bryn Mawr, PA 19010, USA; 2 Département de sciences biologiques, Institut de recherche en biologie végétale, University of Montreal, 4101 East, Sherbrooke Street, Montreal, QC, CanadaH1X 2B2; 3 Ecology Center and Department of Watershed Science, Utah State University, Logan, UT 84322, USA; 4 Smithsonian Environmental Research Center, PO Box 28, 647 Contees Wharf Road, Edgewater, MD 21037, USA

**Keywords:** Conspecific, global change, invasive, nitrogen, nitrogen productivity, phenotypic plasticity, relative growth rate, specific leaf area, wetland.

## Abstract

Physiological ecology and plant functional traits are often used to explain plant invasion. To gain a better understanding of how traits influence invasion, studies usually compare the invasive plant to a native congener, but there are few conspecific examples in the literature. In North America, the presence of native and introduced genetic lineages of the common reed, *Phragmites australis*, presents a unique example to evaluate how traits influence plant invasion. We reviewed the literature on functional traits of *P. australis* lineages in North America, specifically contrasting lineages present on the Atlantic Coast. We focused on differences in physiology between the lineage introduced from Eurasia and the lineage native to North America, specifically seeking to identify the causes underlying the recent expansion of the introduced lineage. Our goals were to better understand which traits may confer invasiveness, provide predictions of how these lineages may respond to interspecific competition or imminent global change, and provide guidance for future research. We reviewed published studies and articles in press, and conducted personal communications with appropriate researchers and managers to develop a comparative dataset. We compared the native and introduced lineages and focused on plant physiological ecology and functional traits. Under both stressful and favourable conditions, our review showed that introduced *P. australis* consistently exhibited greater ramet density, height and biomass, higher and more plastic relative growth rate, nitrogen productivity and specific leaf area, higher mass specific nitrogen uptake rates, as well as greater phenotypic plasticity compared with the native lineage. We suggest that ecophysiological and other plant functional traits elucidate potential mechanisms for the introduced lineage's invasiveness under current and predicted global change conditions. However, our review identified a disconnect between field surveys, experiments, natural competition and plant ecophysiology that must be addressed in future field studies. Given the likelihood of hybridization between lineages, a better understanding of plant traits in native, non-native and hybrid lineages is needed to manage current invasions and to predict the outcome of interactions among novel genotypes. Comparative physiology and other plant functional traits may provide additional tools to predict the trajectory of current and potential future invasions.

## Abstract (precis)

Biological invasion pose serious threats to biodiversity and ecosystem services worldwide. While the effects of invasive species are well-documents, less is known about which specific plant traits convey “invasiveness” because most studies compare closely related, but different species which can confound results. A review of the literature by Mozdzer and other scientists compared genetic lineages of the same species, those native to North American and a lineage introduced from Europe to address this complex issue. The authors found that the ability to change both physiologically and morphologically were the key to success of the introduced genetic lineage under current and predicted global change conditions.

## Introduction

Plant invasions threaten biodiversity and ecosystem services worldwide (Vitousek *et al*. 1997). Numerous studies have investigated plant invasion by comparing non-native species with closely related native congeners, and subsequently relating plant invasiveness to the differences in plant traits among the species compared (McDowell 2002; Deng *et al*. 2004; Drenovsky *et al*. 2012; Caplan and Yeakley 2013). A potential issue with this approach is that congeneric comparisons can be confounded by phylogenetic differences (Harvey 1996). Although not always possible, the ideal approach for assessing how strongly plant traits contribute to invasiveness would minimize phylogenetic differences, specifically by using conspecific individuals that are present in the same geographic range. In North America, multiple conspecific lineages of the common reed, *Phragmites australis* (hereafter *Phragmites*), co-exist (Saltonstall 2002). This provides a unique opportunity to identify the heritable traits and ecophysiological differences that may contribute to invasion success.

Cosmopolitan in distribution (Haslam 1972), *P. australis* is one of the most studied wetland plants due in part to its perceived benefits and threats to ecosystem services. In North America, *Phragmites* is often considered a nuisance species (but see Kiviat 2013) as invasion results in a loss of habitat (Chambers *et al*. 1999; Weinstein and Balletto 1999), reductions in species richness and diversity (Chambers *et al*. 1999; Bertness *et al*. 2002) and alterations to biogeochemical cycles (Windham and Lathrop 1999; Meyerson *et al*. 2000; Windham and Ehrenfeld 2003). Elsewhere, *Phragmites* is either managed or preserved for shoreline stabilization (Benner *et al*. 1982), faunal habitat (Poulin *et al*. 2002) or building materials (Haslam 2010). It is also an important species in wetland-based wastewater treatment systems (Vymazal *et al*. 2006; Brisson and Chazarenc 2009).

*Phragmites australis* consists of dozens of distinct genetic lineages (Saltonstall 2002), seven of which are found in North America (Saltonstall 2002; Meyerson *et al.* 2012). While the genus *Phragmites* has a history of gene flow (Lambertini *et al*. 2012), North American genetic lineages have been geographically separated for millennia. The relatively recent introduction of the Eurasian lineage (haplotype M) most likely occurred in the 19th century (Saltonstall 2002). Historically, the North American native subspecies (*P. australis* subsp. *americanus*; hereafter ‘native *Phragmites*’) (Saltonstall 2002) was considered to be a minor component of both tidal and non-tidal wetlands throughout North America (Marks *et al*. 1994; Chambers *et al*. 1999). The cryptic invasion of *P. australis* subsp*. australis*, or haplotype M (hereafter ‘introduced *Phragmites*’), threatens a wide range of habitats across North America, including tidal fresh wetlands (Rice *et al*. 2000), brackish wetlands (Windham and Lathrop 1999; McCormick *et al*. 2010*b*), salt marshes (Silliman and Bertness 2004), fens (Richburg *et al*. 2001), roadside ditches (Brisson *et al*. 2010) and freshwater coastal wetlands (Tulbure *et al*. 2007; Tulbure and Johnston 2010). Recent work has also identified four additional lineages of *Phragmites* along the North American Gulf Coast, including a hybrid between the Gulf Coast native lineage (*P. australis* subsp. *berlanderii*) and the introduced Eurasian lineage (Lambertini *et al*. 2012).

The presence of conspecific lineages of *Phragmites* along the Atlantic Coast of North America provides a unique opportunity to identify the heritable traits that confer success to invasive plants. Past research has demonstrated that multiple introductions of *Phalaris arundinacea* resulted in increased genetic variation and contributed to invasion in the introduced range (Lavergne and Molofsky 2007). Earlier studies of *Phragmites* in Europe identified population- and/or clone-specific differences in plant phenotype and physiological traits (Rolletschek *et al*. 1999; Lessmann *et al*. 2001; Hansen *et al*. 2007). However, until recently, it was not possible to attribute these differences to a particular genetic lineage. Current molecular tools now provide a framework to assess ecological questions based on evolutionary history, potential speciation due to geographical separation and/or hybridization (Meyerson *et al*. 2010; Lambertini *et al*. 2012). In North America, the introduced Eurasian lineage (haplotype M) is generally considered to be invasive and responsible for the increased dominance of *Phragmites* throughout the North American wetlands. At the same time, native Atlantic Coast lineages are in decline (Saltonstall 2002). Owing to separations in flowering phenology (which limit hybridization) and lack of intermediate morphological forms (Saltonstall 2003, 2011), intraspecific lineages can be used to understand which plant traits may confer invasiveness.

Physiological plant traits and responses to abiotic conditions can influence the spatial distribution of plants from the species to the population level (Chapin and Oechel 1983; Reich *et al*. 1999; Lavergne and Molofsky 2007). When identifying plant traits that may confer invasiveness, spurious interpretations can be avoided by restricting contrasts to those within genera or species. Previous studies have shown that differences in traits such as maximum photosynthetic rate (*A*_max_) (Lavergne and Molofsky 2007; Mozdzer and Zieman 2010), specific leaf area (SLA) (McDowell 2002; Mozdzer and Zieman 2010) and relative growth rate (RGR) (Vasquez *et al*. 2005) can greatly influence the ability of a plant to be successful under a variety of environmental conditions. Here we use a literature review to identify key differences in plant ecophysiology, intraspecific competition and responses to global change factors that distinguish North American native from introduced lineages (haplotype M) of the common reed, *P. australis*. We also highlight areas of future research necessary to understand interactions in the field with regard to intraspecific and intrageneric competition.

## Methods

We reviewed the peer-reviewed literature and unpublished theses that directly compared native and introduced *Phragmites* lineages, and conducted interviews with individuals involved in *Phragmites* research and management. We only included studies that focused on native and non-native lineages along the Atlantic Coast, where clear genetic differences between the lineages had been demonstrated (Saltonstall 2011). We excluded work prior to 2002 in our review because the native and introduced lineages were typically not differentiated prior to that date. To take into account potential differences in abiotic environment, experimental set-up and differences in propagule source (seed versus rhizome), we relativized data for each trait by calculating the per cent difference between the two lineages. This was specifically calculated as the mean trait value of the introduced lineage minus the mean trait value of the native lineage, divided by the mean trait value of the native lineage, and multiplied by 100. Positive values indicated a greater advantage to the introduced *Phragmites* and negative values indicated a greater advantage to the native *Phragmites*. For data obtained from field studies we calculated mean ramet density (ramets m^−2^), leaf area per ramet (cm^2^ ramet^−1^), ramet height and aboveground biomass (g m^−2^). When published data were available, we also calculated mean SLA (cm^2^ g^−1^) and mean nitrogen productivity (NP; RGR per unit gram of nitrogen).

## Results

### Comparative morphology

While ramet densities varied, mass per ramet and mass on a ground area basis were always greater in the introduced lineage. Introduced *Phragmites* produced from 15 to 191 % more biomass under field conditions and from 69 to 286 % higher biomass under experimentally controlled conditions (Table 1). There were no instances where the native type produced more biomass than the introduced type. Such differences are due to plants being taller under both field (6–30 %) and experimental (14–49 %) conditions (Table 1); i.e. they support a larger photosynthetic canopy (36–38 % under field conditions (Table 1) and 14–314 % under experimental conditions (Table 2)).

**Table 1.** Relative differences in plant trait values between North American Atlantic Coast native and Eurasian introduced *Phragmites* in field studies. Relative difference was calculated as the mean trait value of the introduced lineage minus the mean trait value of the native lineage, divided by the mean trait value of the native lineage, and multiplied by 100.

Variable	Habitat	Site	Relative difference	Citation
Plant density (ramets m^−2^)	Brackish	Choptank River, MD	88	Mozdzer and Zieman (2010)
	Brackish	Choptank River, MD	85	Tulbure *et al*. (2012)
	Brackish	Appoquinimink and St Jones, DE	−23	Meadows (2006)
	Fresh	Lac Saint-François, Canada	−28	J. Brisson *et al.* (unpubl. data)
Plant height (cm)	Brackish	Choptank River, MD	16	Tulbure *et al*. (2012)
	Brackish	Appoquinimink River, DE	30	League *et al.* (2006)
	Brackish	Appoquinimink and St Jones, DE	6	Meadows (2006)
	Fresh	Lac Saint-François, Canada	16	J. Brisson *et al.* (unpubl. data)
Aboveground biomass (g m^−2^)	Brackish	Appoquinimink and St Jones, DE	15	Meadows (2006)
	Brackish	Choptank River, MD	191	Mozdzer and Zieman (2010)
Leaf area (cm^2^ ramet^−1^)	Brackish	Choptank River, MD	38	Mozdzer and Zieman (2010)
	Brackish	Appoquinimink and St Jones, DE	36	Meadows (2006)
Leaf N content (%)	Brackish	Choptank River, MD	28	Mozdzer and Zieman (2010)
	Brackish	Rappahanock River, MD	25	Mozdzer and Zieman (2010)
	Brackish	Rappahanock River, MD	16	Packett and Chambers (2006)
	Brackish	Multiple	21	Saltonstall (2007)
	Fresh	Lac Saint-François, Canada	7	J. Brisson *et al.* (unpubl. data)
Chlorophyll content (mg g^−1^ leaf)	Brackish	Choptank River, MD	143	Mozdzer and Zieman (2010)
Specific leaf area (cm^2^ g^−1^)	Brackish	Rappahanock River, MD	14	Mozdzer and Zieman (2010)

**Table 2.** Relative differences between North American Atlantic Coast native and Eurasian introduced *Phragmites* from manipulative experiments including common garden, transplant and greenhouse studies. Relative difference was calculated as the mean trait value of the introduced lineage minus the mean trait value of the native lineage, divided by the mean trait value of the native lineage, and multiplied by 100. ^a^Total density including expansion tillers from this study was used in this calculation. ^b^Means were not significantly different in the original study.

Variable	Propagule source	Site	Relative difference	Citation
Plant density (ramets experimental unit^−1^)	Rhizome	MD	224	Mozdzer and Megonigal (2012)
	Seed	MD	121	Saltonstall and Stevenson (2007)
	Rhizome	AZ	77	Saltonstall and Stevenson (2007)
	Rhizome	RI	99	Holdredge *et al*. (2010)^a^
Total biomass (g experimental unit^−1^)	Rhizome	MD	265	Mozdzer and Megonigal (2012)
	Seed	MD	286	Saltonstall and Stevenson (2007)
	Rhizome	RI	69	Holdredge *et al*. (2010)
Plant height (cm)	Rhizome	MD	34	Mozdzer and Megonigal (2012)
	Seed	MD	49	Saltonstall and Stevenson (2007)
	Rhizome	AZ		Vasquez *et al*. (2005)
Belowground : aboveground (∼R : S)	Rhizome	MD	89	Mozdzer and Megonigal (2012)
	Seed	MD	46	Saltonstall and Stevenson (2007)
Leaf area (cm^2^ ramet^−1^)	Rhizome	Denmark	14.^b^	Hansen *et al*. (2007)
	Rhizome	MD	314	Mozdzer and Megonigal (2012)
Specific leaf area (cm^2^ g^−1^)	Rhizome	VA	33	Mozdzer and Zieman (2010)
	Rhizome	Denmark	15^b^	Hansen *et al*. (2007)
	Rhizome	MD	33	Mozdzer and Megonigal (2012)

**Figure 1.**
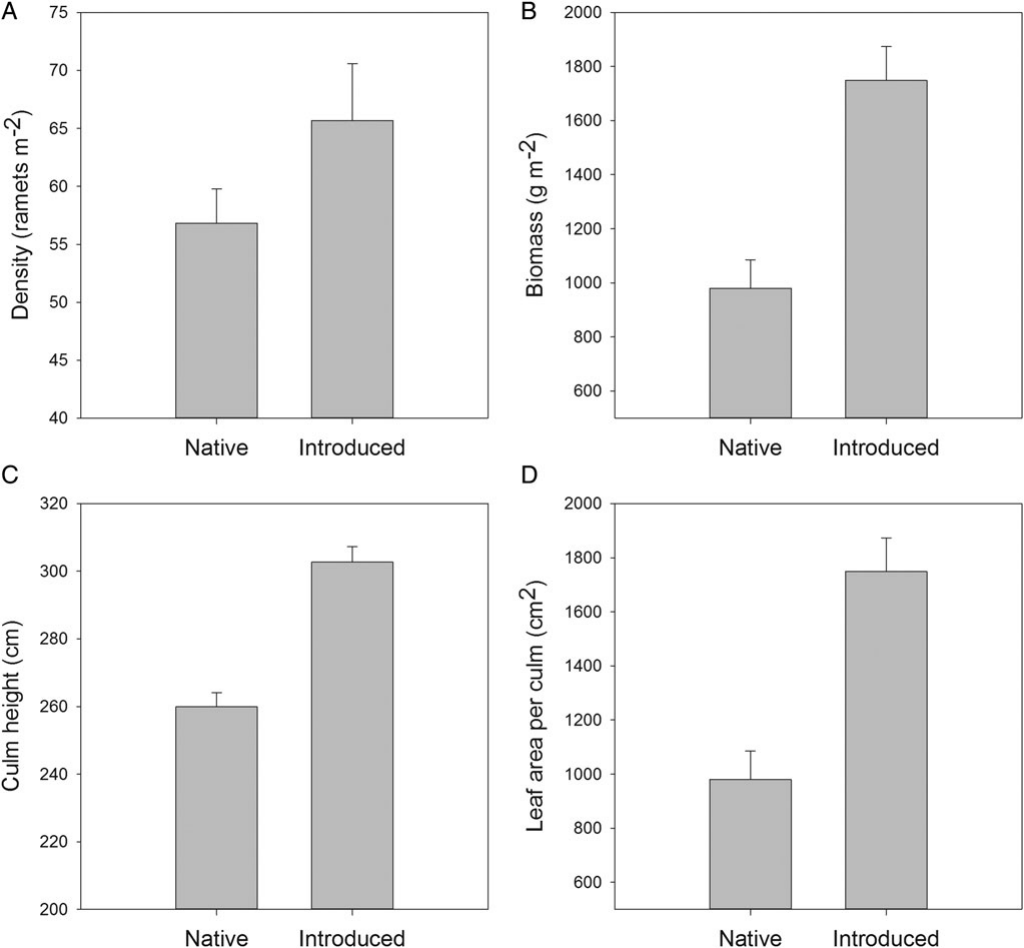
Mean values (±SE) for density (A), biomass (B), culm height (C) and leaf area per culm (D) for *P. australis* lineages native to the North American Atlantic Coast and introduced from Eurasia. All data come from naturally occurring ecosystems. Mean values and standard errors were calculated from the studies that appear in Table 1. The number of studies summarized in (A)–(D) was *n* = 3, *n* = 3, *n* = 6 and *n* = 2, respectively.

Mean ramet densities of the introduced lineage were significantly higher than those of the native lineage (Fig. 1) in both field and experimental settings (Tables 1 and 3), although ramet densities were highly variable for both lineages. Even when the densities of the native and introduced *Phragmites* are similar, ramets of the introduced lineage are most often taller (Table 1, Fig. 1). In the field, ramets were 6–10 % taller, and had a 36–38 % greater leaf area per ramet (Table 1). Density was also greater in introduced versus native *Phragmites* (95–322 %) in growth chamber experiments where carbon dioxide (CO_2_) and nitrogen (N) were manipulated (Table 3). In addition, introduced plants were 13–20 % taller (Table 3) in both field and manipulative experiments.

**Table 3.** Effects of salinity, N and elevated CO_2_ on relative differences between North American Atlantic Coast native and Eurasian introduced *Phragmites* in manipulative field and greenhouse studies*.* Relative difference was calculated as the mean trait value of the introduced lineage minus the mean trait value of the native lineage, divided by the mean trait value of the native lineage, and multiplied by 100. ^a^Means were not significantly different in the original study.

Variable	Propagule source	Treatment	Site	Relative difference	Citation
Density (ramets experimental unit^−1^)	Rhizome	N	MD	168	Mozdzer and Megonigal (2012)
	Seed	N	MD	95	Saltonstall and Stevenson (2007)
	Rhizome	Field + N	RI	100^a^	Holdredge *et al*. (2010)
	Rhizome	Salinity	AZ	873	Vasquez *et al*. (2005)
	Rhizome	CO_2_	MD	322	Mozdzer and Megonigal (2012)
	Rhizome	CO_2_ + N	MD	193	Mozdzer and Megonigal (2012)
Total biomass (g or g m^−2^)	Rhizome	N	MD	171	Mozdzer and Megonigal (2012)
	Rhizome	N	MD	108	Saltonstall and Stevenson (2007)
	Rhizome	Field + N	RI	250	Holdredge *et al*. (2010)
	Rhizome	CO_2_	MD	171	Mozdzer and Megonigal (2012)
	Rhizome	CO_2_ + N	MD	151	Mozdzer and Megonigal (2012)
Plant height (cm)	Rhizome	N	MD	20	Mozdzer and Megonigal (2012)
	Seed	N	MD	16	Saltonstall and Stevenson (2007)
	Rhizome	CO_2_	MD	20	Mozdzer and Megonigal (2012)
	Rhizome	CO_2_ + N	MD	13	Mozdzer and Megonigal (2012)
Belowground : aboveground (∼R : S)	Rhizome	N	MD	100	Mozdzer and Megonigal (2012)
	Seed	N	MD	0	Saltonstall and Stevenson (2007)
	Rhizome	CO_2_	MD	90	Mozdzer and Megonigal (2012)
	Rhizome	CO_2_ + N	MD	54	Mozdzer and Megonigal (2012)
Leaf area (cm^2^ ramet^−1^)	Rhizome	N	MD	201	Mozdzer and Megonigal (2012)
	Rhizome	CO_2_	MD	196	Mozdzer and Megonigal (2012)
	Rhizome	CO_2_ + N	MD	182	Mozdzer and Megonigal (2012)
Specific leaf area (cm^2^ g^−1^)	Rhizome	N	MD	28	Mozdzer and Megonigal (2012)
	Rhizome	CO_2_	MD	13	Mozdzer and Megonigal (2012)
	Rhizome	CO_2_ + N	MD	5	Mozdzer and Megonigal (2012)

### Canopy differences

Phenotypic differences in colour and canopy structure are indicative of physiological differences. Native *Phragmites* is characteristically yellow–green in colour, whereas the introduced lineage is more blue–green in colour throughout North America (Blossey 2002; Mozdzer and Zieman 2010; Swearingen and Saltonstall 2010). In Atlantic Coast populations, the characteristic yellow–green colour of the native lineage was related to it having 143 % lower chlorophyll content and 14 % thicker leaves (lower SLA) (Table 1) than the introduced lineage (Mozdzer and Zieman 2010). We report anywhere from 12 to 80 % lower light-saturated rates of photosynthesis (*A*_max_) (Table 4) than the introduced population due to lower chlorophyll content and lower SLA (Mozdzer and Zieman 2010) translating into the observed lower RGR (Vasquez *et al*. 2005; Mozdzer and Megonigal 2012). Given the consistently observed phenotypic differences among North American native populations, it is likely that differences in photosynthetic physiology are similar across North American native populations.

**Table 4.** Relative physiological differences between North American Atlantic Coast native and Eurasian introduced *Phragmites.* Relative difference was calculated as the mean trait value of the introduced lineage minus the mean trait value of the native lineage, divided by the mean trait value of the native lineage, and multiplied by 100. ^a^Trait means were not significantly different in the original study. ^b^NP was estimated from published data.

Variable	Experiment type	Treatment	Site	Relative difference	Citation
N uptake rate (µmol g^−1^ h^−1^)	Lab	NH_4_	VA	50	Mozdzer *et al*. (2010)
	Lab	Urea-N (DON)	VA	0	Mozdzer *et al*. (2010)
	Lab	Glycine (DON)	VA	30^a^	Mozdzer *et al*. (2010)
	Lab	Glutamic acid (DON)	VA	28^a^	Mozdzer *et al*. (2010)
Nitrogen productivity (g gN^−1^ day^−1^)	Chamber	Control	MD	118	Mozdzer and Megonigal (2012)
	Chamber	N	MD	26	Mozdzer and Megonigal (2012)
	Chamber	CO_2_	MD	81	Mozdzer and Megonigal (2012)
	Chamber	CO_2_ + N	MD	111	Mozdzer and Megonigal (2012)
	Garden	Control (0.02 M)	AZ	21	Vasquez *et al*. (2005)^b^
	Garden	Salinity (0.17 M)	AZ	34	Vasquez *et al*. (2005)^b^
Leaf : root GS activity	Field	None	ME	12	Hazelton *et al*. (2010)^b^
*A*_max_ (µmol CO_2_ m^2^ s^−1^)	Field	None	MD	33	Mozdzer *et al*. (2010)
	Greenhouse	None	VA	80	Mozdzer *et al*. (2010)
	Garden	None	Denmark	12^b^	Hansen *et al*. (2007)
Relative growth rate (g g^−1^ day^−1^)	Chamber	Control	MD	116	Mozdzer and Megonigal (2012)
	Chamber	N	MD	30	Mozdzer and Megonigal (2012)
	Chamber	CO_2_	MD	57	Mozdzer and Megonigal (2012)
	Chamber	CO_2_ + N	MD	36	Mozdzer and Megonigal (2012)
	Garden	Control	AZ	10	Vasquez *et al*. (2005) (0.02 M)
	Garden	Salinity	AZ	25	Vasquez *et al*. (2005) (0.13 M)
Ventilation efficiency (mL min^−1^ Pa^−1^ m^−2^)	Field	None	MD	320	Tulbure *et al*. (2012)

**Figure 2.**
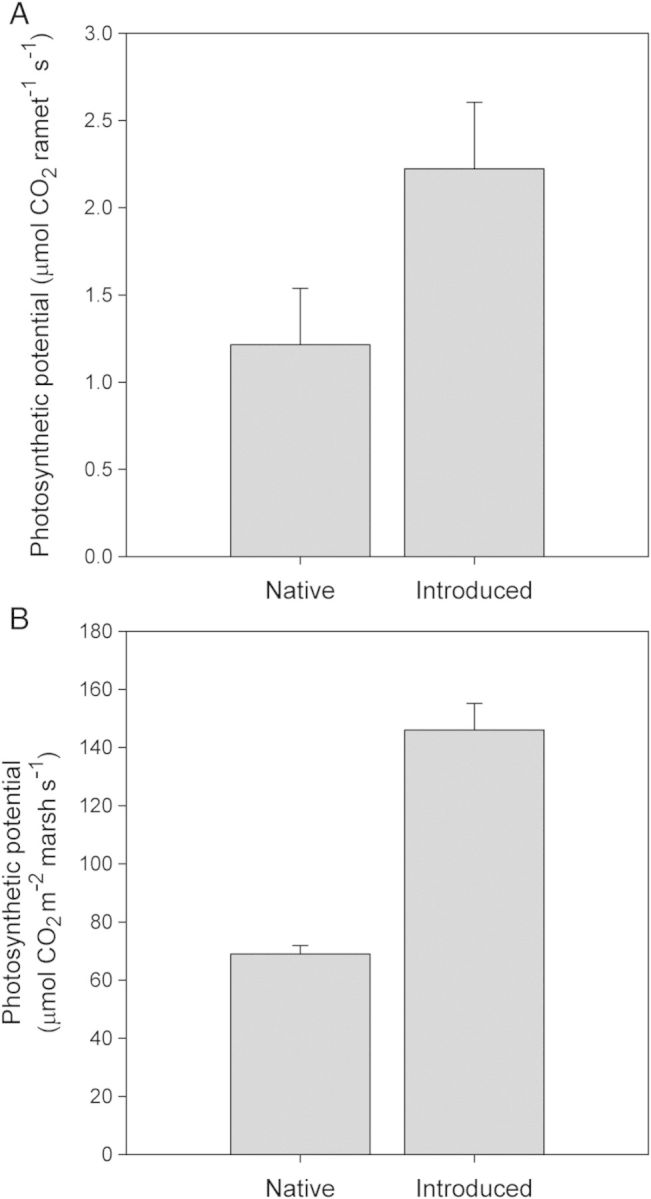
Estimated CO_2_ assimilation rate (A) per ramet and (B) per unit ground area of both North American Atlantic Coast native and Eurasian introduced *Phragmites.* Photosynthetic potentials were estimated from the mean trait values that appear in Tables 1 and 4.

Investment in both light-harvesting capacity (leaf area ramet^−1^) and fast growth (SLA and RGR) differentiates the two lineages. The introduced lineage had a 14–33 % greater SLA, and this difference in SLA is consistent among populations for plants grown under field experimental conditions (Tables 1 and 2). Consistent with theory (Ceulemans 1989; Westoby 1998), increased SLA also corresponds to higher RGRs (10–116 %; Table 2) of the introduced lineage under current and predicted elevated CO_2_ and N pollution conditions. In addition, on a per ramet basis, introduced *Phragmites* had anywhere from 36 % to over 300 % greater leaf area than the native type (Tables 1 and 2). While both lineages have high photosynthetic rates (Mozdzer and Zieman 2010), the introduced lineage has anywhere from 12 to 80 % greater rates of photosynthesis per unit leaf area (Table 4). To illustrate the potential ecological significance of these photosynthetic rates on potential plant growth, we used data on mean ramet density, mean size of the photosynthetic canopy and mean photosynthetic rates (Table 1 and Fig. 1) to calculate stand-scale photosynthesis rates. Assuming full light penetration to all leaves on an individual plant, we found that the introduced lineage would fix 83 % more CO_2_ per ramet per second (Fig. 2) than the native lineage. By taking into account the *Phragmites* density per unit area, our analysis suggests that introduced *Phragmites* has the potential to fix 112 % more C on a stand scale than native *Phragmites* (Fig. 2). These differences are compounded by phenological differences in senescence, as the introduced lineage has the potential to photosynthesize for weeks to months longer than the native lineage (Meyerson *et al*. 2010; Mozdzer and Zieman 2010). Congruent with greater carbon fixation potential and higher SLA, the introduced lineage consistently exhibited a greater RGR (Table 1) than the native lineage under a range of conditions (Vasquez *et al*. 2005; Mozdzer and Megonigal 2012).

### Belowground

Only a few studies have investigated belowground differences between native and introduced *Phragmites*, yet trait differences associated with belowground allocation have the potential to magnify differences in growth potential. The non-native lineage had a greater ratio of belowground : aboveground biomass, allocating 46–89 % more biomass belowground both proportionally and in absolute terms under ambient nutrient conditions (Table 2). The belowground : aboveground ratio was not significantly different under N treatment when plants were grown from seed (Table 3), but when grown from rhizomes, the introduced lineage allocated 54–100 % more biomass belowground than did the native lineage (Table 3). Of this belowground allocation, Mozdzer and Megonigal (2012) reported that the introduced lineage allocated proportionally more biomass to both roots (root mass fraction) and rhizomes (rhizome mass fraction) than the native lineage. Thus, higher rates of nutrient acquisition and clonal expansion may come from greater resource allocation belowground to both rhizomes and roots.

### Nutrient uptake, plant N demand and N metabolism

A study comparing the partitioning of glutamine synthetase (GS) activity, a proxy for nitrogen use efficiency (NUE) (see reviews by Oaks 1992; Andrews *et al*. 2004), demonstrated that the leaf/root partitioning of GS activity of a *Phragmites*-dominated habitat was the highest recorded in a natural system. Although there was no significant difference between *Phragmites* lineages, both had among the highest leaf/root GS activity measured in land plants, scoring higher than transgenic plants that were modified to express this trait (Hazelton *et al*. 2010). The comparably high NUE was reflected in several studies that have shown similar vigour and assimilation of N at low concentrations (Holdredge *et al*. 2010; Mozdzer *et al*. 2010; Mozdzer and Megonigal 2012). Both lineages have higher affinities for ammonium when compared with dominant tidal wetland plants and both use multiple forms of organic N. *Phragmites australis* may therefore have access to a pool of nutrients that is not used by competing plants (Mozdzer *et al*. 2010). While both lineages have high affinities for N, native *Phragmites* has a higher affinity for NH_4_^+^, but uptake rates saturate at a lower N concentration (Mozdzer *et al*. 2010). Thus, under low nutrient conditions, both lineages would be expected to perform equally well (Holdredge *et al*. 2010; Mozdzer *et al*. 2010). However, as anthropogenic N loading increases, the advantage clearly shifts to introduced *Phragmites* (Holdredge *et al*. 2010; Mozdzer *et al*. 2010), as demonstrated by the greater vigour relative to the native lineage for all measured traits and metrics (Table 3).

Mozdzer and Megonigal (2012) found that only the introduced lineage, and not the native lineage of *Phragmites*, can alter its N metabolism to match a variety of N availability conditions. In particular, under low N availability, the introduced lineage changes plant NP, an integrative term of nutrient use efficiency, dramatically altering N metabolism to match growing conditions. In contrast, the native lineage has a nearly static NP for low-N environments. Data from the Vasquez *et al*. (2005) study reveal the same pattern (Table 4), with the introduced *Phragmites* exhibiting a greater NP under ambient and high-salinity conditions.

### Global change effects

The most striking differences between the North American native and introduced lineages are when they are experimentally exposed to global change factors such as anthropogenic N pollution, elevated CO_2_ or salinity. In particular, introduced *Phragmites* had a greater physiological and morphological plasticity under both stressful and resource-rich conditions, resulting in its designation as a ‘Jack-and-master’ strategist (Mozdzer and Megonigal 2012). Because of this greater plasticity, introduced *Phragmites* had a greater density with added N (85–168 %), salinity (873 %) and elevated CO_2_ (193–322 %); introduced plants are 13–20 % taller and have 182–201 % greater leaf area per ramet (Table 3). As a consequence of increased density, height and leaf area, the introduced lineage produced anywhere from 151 to 250 % more total biomass (aboveground + belowground) (Table 3). Of the biomass produced, the introduced lineage allocated 54–100 % proportionally more belowground (Table 3).

## Discussion

### Physiological ecology and invasiveness of the introduced *Phragmites*

Our review confirms that introduced and native *Phragmites* lineages differ both physiologically and morphologically. Introduced plants are generally taller and occur in greater densities, which results in greater productivity in the introduced lineage in nearly every study. The taller and denser canopies (Meadows 2006; Mozdzer and Zieman 2010; Mozdzer and Megonigal 2012) and thick litter layer (Holdredge and Bertness 2011) in stands of the introduced lineage cumulatively result in reduced light availability. The introduced *Phragmites* may also transmit oxygen to rhizomes and roots more efficiently (Tulbure *et al*. 2012), a feature that would potentially give it a belowground competitive advantage by ameliorating the anaerobic rhizosphere of saturated soils. As a consequence of its greater biomass, introduced *Phragmites* may be more effective at immobilizing N; thus it may limit the N available to competitors (Meyerson *et al*. 2000; Windham and Meyerson 2003) or facilitate invasion through competitive exclusion (Holdredge and Bertness 2011).

The higher ramet density of the introduced lineage, observed in both field and experimental settings, suggests differences in clonal strategies. The introduced lineage initially spreads through guerilla growth, sending out individual stolons. It then transitions to phalanx growth, resulting in the formation of dense patches that exclude other vegetation (Windham and Lathrop 1999; Amsberry *et al*. 2000). In contrast, the native lineage does not always exhibit phalanx growth, as demonstrated by the fact that native *Phragmites* stands are interspersed with other species (E. L. G. Hazelton and V. Douhovnikoff, pers. comm.). The production of a greater number of tillers by the invasive lineage results in a higher ramet density and biomass per unit area, which thereby increases its potential for invasion (Holdredge *et al*. 2010).

Given the consistent phenotypic differences in North American native populations, we hypothesize that differences in photosynthetic physiology are similar across North American native populations. We base this on the fact that the native population has lower *A*_max_ rates compared with the introduced population, which is due to lower chlorophyll content and lower SLA (Mozdzer and Zieman 2010) translating into a lower RGR (Vasquez *et al*. 2005; Mozdzer and Megonigal 2012). More common garden and field studies are needed, especially across multiple populations and study sites, to validate this observation with regard to potential differences in chlorophyll content, accessory pigments and SLA.

Increased light-harvesting capacity (leaf canopy per ramet) and higher growth rates (SLA and RGR) are indicative of underlying physiological strategies. In particular, the greater and plastic SLA and higher RGR of introduced *Phragmites* have been suggested as factors driving its invasion (Mozdzer and Zieman 2010; Mozdzer and Megonigal 2012). Although leaf-level photosynthetic rates respond immediately to local environmental conditions (Lessmann *et al*. 2001), traits such as SLA, which combine physiological and biochemical processes, are slower to respond (Callaghan *et al*. 1992) and are excellent predictors of potential plant growth (Ceulemans 1989). While the lower SLA of the native lineage should confer some resistance to herbivory, herbivory by invertebrates seems to be greater on native populations (Lambert and Casagrande 2007; Lambert *et al*. 2007), suggesting that the decreased SLA did not evolve for herbivory defence. Lower SLA could be attributed to an adaptation for slower growth under nutrient-limited conditions, where plants invest more in longer-lived structures.

The greater resource allocation belowground (to both rhizomes and roots) in the introduced lineage may result in both higher rates of nutrient acquisition and high rates of clonal expansion, contributing to both growth and clonal expansion. Historically, clonal integration and resource sharing were prominent hypotheses used to explain the invasiveness of introduced *Phragmites* (Amsberry *et al*. 2000). However, given the recent findings of high within-patch genetic diversity (McCormick *et al*. 2010*a*, *b*), and different potential growth strategies between native and introduced *Phragmites* (E. L. G. Hazelton and V. Douhovnikof, unpubl. data), more research is needed to conclusively determine the importance of resource sharing, and whether there are differences among native and introduced lineages. Resource sharing and a greater ability to efficiently exchange gases between aboveground and belowground organs (Tulbure *et al*. 2012) may provide a mechanism to facilitate establishment and expansion in environments such as salt marshes that have pronounced stress gradients and limit plant distributions.

Our review showed that both *Phragmites* lineages are adapted to N-limited environments, and that both lineages have a similar high-affinity transport system, which is an adaptation to N limitation (Crawford and Glass 1998). However, the difference in performance under high N indicates that the introduced lineage may be shifting to a more efficient low-affinity transport system than the native lineage. The ability to respond to changing nutrient conditions has been suggested as one of the competitive advantages of the introduced *Phragmites*, while the native lineage becomes N saturated and is not able to exploit eutrophic conditions (Mozdzer *et al*. 2010). Yet, the introduced *Phragmites* is not at a complete disadvantage in low-N environments, due to its plastic N productivity (Mozdzer and Megonigal 2012). These studies indicate that the vigour of introduced *Phragmites* will increase with anthropogenic nutrient pollution, and provide evidence that the competitive ability of introduced *Phragmites* may be linked to plastic nutrient use strategies under lower nutrient availability.

Taken together, the physiological and other functional trait advantages of the introduced lineage (greater density, ramet height and biomass, higher RGR and SLA, and high N uptake under high anthropogenic N loading) are major factors driving its invasiveness in North America.

### Competition between native and introduced *Phragmites*

The overall superior performance of introduced *Phragmites* suggests that it would outcompete the native *Phragmites* in mixed populations. Indeed, the increase in abundance of introduced *Phragmites* with the concomitant decrease in the native one at the landscape scale is often interpreted as being the result of direct competition (Saltonstall 2002; Lelong *et al*. 2007). However, processes other than competitive exclusion may result in similar patterns. For example, a disturbance causing the removal of native *Phragmites* may facilitate the establishment of the introduced lineage. In such cases, better dispersal, establishment and expansion of introduced *Phragmites*, and not direct resource competition, would be responsible for the observed shift in relative abundance at the landscape scale.

If competitive exclusion occurs, the most direct field evidence would come from the contact zone of adjoining native and introduced stands. Competitive outcomes would be revealed by the spatial dynamics at that contact zone over time as one lineage progresses to the detriment of the other. Such studies remain rare, and their results are inconclusive or contradictory. In a study in the Lac Saint-François Reserve of southern Quebec, five contact zones of neighbouring stands growing in freshwater wetlands were surveyed for up to 5 years (S. de Blois *et al.*, unpubl. data). The survey did not reveal a clear pattern of progression of the introduced over the native lineages, or that the introduced patches were increasing over the course of the survey. Instead, there were variations in progression or regression between sites and between years, with only a slight (and non-significant) net advantage for the introduced lineage. Meadows (2006) surveyed five transects crossing the contact zones in each of two cases of adjoining stands of native and introduced *Phragmites* in Delaware. During the 2 years of the survey, there appears to have been an increase in the relative density of the native lineage over the introduced lineage in the ‘mixed’ zone of one site and a small decrease in the native lineage at the other site, although interannual changes in density for either lineage were not significant. Meadows (2006) also measured the expansion rate of adjoining stands of native and introduced *Phragmites* located in a different Delaware site. Comparing the position of the most distant culm outside the leading edge of the stands positioned the previous year, he found that the introduced stand expanded by 1.11 m, while the adjoining native stand contracted or was displaced by 1.59 m.

Classical garden or greenhouse competition experiments using seedlings or transplants, with various combinations of mixed and pure populations, represent the most direct approach to evaluate competitive interaction between two plant species (Gibson *et al*. 1999; Holdredge *et al*. 2010). We found one such study in our review; Holdredge *et al*. (2010) transplanted native and introduced *Phragmites* plants to a common field, and manipulated both the identity of competitors and fertilization. Although they found no evidence of suppression of the native lineage after 2 years, their results suggest that, under high-nutrient conditions, the invasive lineage would displace the native lineage over time by producing more biomass and expanding at a faster rate.

In a mesocosm competition experiment, S. de Blois *et al.* (unpubl. data) compared the expansion of native or introduced *Phragmites* grown in one half of the mesocosms into the opposite, competitor-occupied half, as well as expansion into mesocosms with unoccupied (control) halves. While the absolute performance of introduced *Phragmites* in terms of biomass and ramet density was superior to the native one under all circumstances, there was no statistical difference in the overall percentage of decrease in performance caused by the presence of the competitor. For example, expansion into the opposite compartment 1 year after a central panel was removed, as measured by aboveground biomass, was approximately 65 % lower for both subspecies in competition mesocosms compared with the control. By producing more biomass and a larger number of culms, the results nonetheless suggest that the relative competitive effect of the introduced *Phragmites* on the native one would increase over time. Because a decline in the native lineage has been related to an increase in the introduced lineage, there is still a need for more experimental research on competition between the lineages in order to clarify the conditions that may lead to competitive exclusion.

### Responses to global change factors (anthropogenic N pollution and CO_2_)

Our review finds that introduced *Phragmites* is a ‘Jack-and-master’ of change, which is a similar characterization to that of a super weed (Baker 1965). In other words, the introduced lineage outperforms the conspecific native lineage under both stressful and resource-rich conditions. Inherently higher RGRs, greater and plastic SLA, and plastic NP are suggested to be the physiological mechanisms unique to the introduced lineage that enhance its invasive ability under current and future conditions (Mozdzer and Megonigal 2012). More research is needed to elucidate the reasons behind the greater plasticity and ecological fitness of introduced *Phragmites*. Whether its plasticity and fitness are related to a history of multiple introductions (Hauber *et al*. 2011), hybridization (Freeland *et al*. 2010; Meyerson *et al*. 2010; Lambertini *et al*. 2012) or evolution of increased competitive ability (Blossey and Notzold 1995) is still unclear (but see Guo *et al.* 2013). This focus area would greatly benefit from an investigation of heritable changes in gene expression via an epigenetic approach (Nicotra *et al*. 2010).

Our literature survey suggested that introduced *Phragmites* will continue to expand its range and become more abundant in response to continuing change in the global environment. In particular, anthropogenic N pollution benefits the introduced lineage; it has a stem density that is 181 % higher, produces 85–171 % more biomass and has ramets that are 13–20 % taller under elevated N (Table 3). In addition, N had profound effects on the introduced lineage by producing a canopy with 200 % greater photosynthetic area (Table 3). These differences in growth can be attributed to the greater N uptake capacity of the introduced lineage (Mozdzer *et al*. 2010) coupled to a greater allocation belowground for nutrient acquisition (Tables 2 and 3). Plastic NP (Mozdzer and Megonigal 2012) may be the underlying cause for the disproportionate response under current and predicted N availabilities. This is congruent with correlations of introduced *Phragmites* expansion throughout New England (Bertness *et al*. 2002) and Chesapeake Bay (King *et al*. 2007; Chambers *et al*. 2008) with anthropogenic N pollution.

As C_3_ plants, both *Phragmites* lineages should benefit from elevated CO_2_ (Ainsworth and Long 2005). In growth chamber experiments (Mozdzer and Megonigal 2012), both lineages responded positively to elevated CO_2_. However, the introduced lineage had the greatest biomass response to CO_2_, which was about 45 % greater than the control treatment. This suggests, but does not demonstrate, that it is likely that elevated CO_2_ will also favour the introduced genetic lineage in the field. Elsewhere, only a handful of studies have investigated CO_2_ responses in *Phragmites.* Neither the growth chamber study on *Phragmites japonica* or *Phragmites communis* (Kim and Kang 2008) nor field experiments with *Phragmites* within a *Sphagnum* peatland (Milla *et al*. 2006) demonstrated any significant effects of elevated CO_2_ on *Phragmites* growth. It is most likely that the elevated CO_2_ growth response in Kim and Kang's (2008) study was limited by pot volume, which is a well-documented phenomenon (Thomas and Strain 1991). A mini-FACE experiment in Europe by Milla *et al*. (2006) concluded that vascular plants in peatlands, including *Phragmites*, are not very responsive to elevated CO_2_. The lack of CO_2_ response by *Phragmites* in the mini-FACE study was likely attributable to the CO_2_ concentration at the position of the tall *Phragmites* canopy being close to ambient levels and/or a combination of nutrient limitation and immobilization by the *Sphagnum* layer (Milla *et al*. 2006). Alternatively, it is also possible that the introduced *Phragmites* lineages in North America are physiologically different from those in Eurasia.

In short-term studies, rising CO_2_ and anthropogenic N pollution seem to benefit the introduced lineage with respect to both expansion and establishment. In particular, the introduced lineage outperformed the native lineage for every measurable metric (Table 4); the introduced lineage exhibited a more plastic NP and SLA and an inherently higher RGR (Richburg *et al*. 2001) The introduced lineage also exhibits a ‘Jack-and-master’ phenotypic and physiological plasticity (*sensu* Richards *et al*. 2006), suggesting that it had greater ecological fitness under both stressful and resource-rich conditions. These results suggest that the introduced lineage will only become more competitive in the future.

## Conclusions

Given the high genetic diversity within native and introduced *Phragmites* populations (McCormick *et al*. 2010*a*; Saltonstall 2011), the underlying question is what caused the introduced lineage to become so invasive in North America? Our review clearly identifies gaps in our knowledge. Additional studies are needed to determine whether there has been an evolution of increased competitive ability (Blossey and Notzold 1995) given potential physiological differences between North American and Eurasian populations. An alternative explanation is that there has been gene flow among North American native and introduced populations that made the introduced lineage more invasive and/or plastic than it is outside of North America. Given the amount of gene flow recently demonstrated in Gulf Coast populations (Saltonstall 2011; Lambertini *et al*. 2012), and the discovery of new genetic lineages (Lambertini *et al*. 2012), this possibility should be further evaluated.

Finally, our review shows that direct studies of competitive interactions between the native and the introduced *Phragmites* are few, and that conclusions from the laboratory and field observations do not always concur. The assumed superiority of introduced *Phragmites* does not necessarily hold in mixed or adjoining populations under pristine conditions, and inconclusive or even opposing results have occasionally been observed. Certainly, more experiments or surveys of adjoining populations are necessary to examine how physiological and morphological characteristics translate into a competitive advantage of the introduced lineage over the native *Phragmites* when they are naturally co-occurring. Acknowledging the disconnect between laboratory and field observations, we still observe profound differences in response to global change factors such as CO_2_ and N pollution. Thus, our analysis of comparative ecophysiology and functional traits allows us to predict its likely trajectory. Given the differential response of native and introduced *Phragmites*, we hypothesize that the competitive advantage will shift to more strongly favour the introduced lineage, especially when competition is coupled with anthropogenic N pollution and rising CO_2_.

## Sources of Funding

This manuscript is an outcome of a *Phragmites* symposium at the 2011 meeting of the Society of Wetland Scientists with travel funding provided by *AoB PLANTS* to both T.J.M. and E.L.G.H. Funding support for T.J.M. was provided from MD Sea Grant Award SA7528114-WW and NSF DEB-0950080. E.L.G.H. was funded by NOAA NA09NOS4780214, a Smithsonian Predoctoral Fellowship, Utah State University Ecology Center, the Society of Wetland Scientists and Delta Waterfowl.

## Contributions by the Authors

T.J.M. performed the meta-analysis of the published studies. T.J.M., J.B. and E.L.G.H. contributed to the interpretation and writing of the manuscript.

## Conflicts of Interest Statement

None declared.
